# Hospital and Institutional News

**Published:** 1917-04-28

**Authors:** 


### HOSPITAL AND INSTITUTIONAL NEWS.

### THE EXTENSION OF HOME MILITARY HOSPITALS.

The official announcement from the War Office that in consequence of the sinking of hospital ships it is proposed to retain in France a larger proportion of our sick and wounded than hitherto, has resulted in a widespread feeling of uneasiness amongst those responsible for proposed or actually initiated extensions to Red Cross hospitals in this country. In a recent letter to the *Times*, a doctor voiced this anxiety very forcibly and succinctly, and asked for an immediate official pronouncement on this exceedingly important point. So far as we know, at the moment of going to press, no such official pronouncement has been issued, but there is a very prevalent impression, which is probably founded on acquaintance with the views held at the War Office, that there is no intention at present to close down Red Cross hospitals in this country, or to interrupt the influx of military patients to their wards. What is contemplated, it is thought, is that a considerable number of new hospitals will be started in France which would, but for the hospital ship outrages, have been started in this country. A large proportion of the wounded, therefore, will be kept in France, but the numbers sent to England need not be diminished if the casualties continue to mount at the rate they have been doing lately. Incidentally, the wounded will probably stay longer in France than has been customary, so that the work in any individual hospital there will be more interesting to both doctors and nurses, who at present lose sight of their patients so rapidly as to deprive them of much of the interest of their work.

### THE CLOSING OF CIVILIAN HOSPITALS IN LONDON.

It is current gossip in institutional circles that the Government are putting pressure on certain civilian hospitals to close down in order to release more medical men for service with the Army. It is said, apparently on good authority, that those who are interested in the management of gynæcological clinics in London are being asked either to close the gynæcological departments of general hospitals, or to close some of the special gynæcological hospitals. Much as it is to be deplored that any of London's institutional facilities for the sick should be curtailed, it is certainly possible to adduce arguments in favour of this particular procedure. In the first place, a large proportion of those surgeons who are marked down as "indispensable" to these gynæcological clinics, whether at special or general hospitals, are young men of military age. Next, it must be admitted that a considerable percentage of the operations now so plentifully undertaken therein are operations of convenience, not of necessity. Many of the curettings and many of the perineorrhaphies can well wait a year or two until the war is over, rather than that our wounded should go short of necessary surgical skill. Some of the numerous hysterectomies can also be postponed without serious hardship to the patients, as can many of the ventro-suspensions. A good many of the salpingectomies are also postponable; indeed, it might do some young surgeons good to see for themselves that a considerable proportion of pyosalpinx cases will get well of themselves, though few are at present afforded the opportunity of doing so. Ovariotomies, of course, must continue. So far we have not heard what decisions have been arrived at; probably some of them will be made public shortly.

### HOW THE GENERAL HOSPITALS STAND.

In view of the situation thus created, we have tested the prevailing currents of expert opinion as they are setting at the present time in the large voluntary general hospitals of London. There seems to be practical unanimity that to reduce medical staffs still further is hardly possible, if the institutions are to be carried on as hitherto. Some administrators make the reservation that if such young surgeons as they still retain are conscripted, they can manage to serve the civilian population provided older men are available as substitutes. One high official of a very large general hospital would even go so far as to close some of the smaller general hospitals, leaving the larger hospitals to do the work. Another suggestion which has been made is that the smaller V.A.D. and auxiliary hospitals should be closed, on the ground that they are inefficient and wasteful of medical staff. The standard of efficiency amongst auxiliary hospitals throughout the country admittedly varies; but as far as our own observations go, the average level in London is remarkably high, and the arguments upon which this particular proposal is based strike us as betraying unfamiliarity with the actual work and conditions of these hospitals. During the next few days, it is understood, there will be numerous meetings of hospital committees to discuss the new situation; and opinion will no doubt have crystallised, before the next issue of The Hospital, to a greater extent than it can be said to have done at present.

### MILITARY HOSPITAL EFFICIENCY AND ARMY REQUIREMENTS.

The question as to whether the non-medical staffs at military hospitals necessary for the proper working of the institutions should join the Army has been referred to the local Tribunals by the War Office. Previously they have been exempted by the War Office, but these concessions have now been withdrawn in all cases of men of military age. Extreme difficulties have been experienced on every hand to obtain the services of really competent stokers and engine-drivers at military hospitals, and attempts to replace men called to the Colours have in many cases failed. The secretary of King's College Hospital had to appear before the Lambeth Tribunal for permission to retain the services of six of his staff mainly engaged in the mechanical work of the hospital. A letter was read from the War

Office stating that it was not suggested that the work undertaken by the hospital was not of national importance, indeed the Director-General of the Medical Service had notified the Director of Recruiting that the work undertaken by the hospital in connection with the treatment of Army cases was of great importance. Two drivers of the Diesel engines were amongst those whom the hospital authorities especially desired to retain, as on the perfect running of these engines depended the lighting, medical treatment, and everything on which the administration depended for the proper treatment of the wounded soldiers. Despite the fact that the secretary informed the Tribunal that if they lost one of these drivers they would have to report to the War Office that they could not undertake the responsibility of the work, the Tribunal decided in one case only to allow exemption till May 1 final, and in the other three months' exemption.

### A HOSPITAL PRESIDENT'S CURIOUS PESSIMISM.

If Mr. Dyson Perrins, president of the Malvern Hospital, has studied the article on "Voluntary Hospital Finance" in last week's issue of The Hospital, we believe that he will wish to revise the pessimistic conclusions which he drew in the course of the same week in a speech to his hospital's subscribers. In a very few years, he said, the general hospitals will be supported out of Imperial revenues, a change, however, beset with difficulties to which public opinion is "hardening." From a man of Mr. Perrins' experience and habit this lugubrious outlook was not to be expected. It comes almost like a snub from an old friend. For, if we may say so, Mr. Perrins' practice has been much better than his theory. He has conducted the Malvern Hospital with care and enthusiasm, and he is known to be its great benefactor. Only lately he has given £250 to clear off two years' deficits. Since the war has not sapped his constancy and generosity, may there not be others equally immune to change? Since, in spite of taxation, War Loans, and high prices, the hospitals have received more support than before, while £6,000,000 and over has been subscribed to Red Cross Funds, the nation's wealth and generosity seem rather in the nature of a discovery than of exhaustion. When we remember that giving is largely a matter of habit we think that, now this habit has been made so general, the outlook for the voluntary hospitals is cheerier, and more assured than ever. We invite Mr. Perrins to controvert this statement if he can, and we have relieved his modesty by mentioning ourselves his own generosity as a case in point against him!

### A BIG MEMORIAL SCHEME AT NOTTINGHAM.

The speech of Mr. W. G. Player, who has been elected for the third year in succession to the presidency of the Nottingham General Hospital, at the recent annual meeting disclosed the fact that there were probably 1,000 patients awaiting admission to the hospital. He said that he hoped a great effort would one day be made to increase the number of beds. This hint was at once taken up by Mr. James Forman, who proposed that the time was ripe to consider a permanent memorial to the fallen soldiers "by placing the hospital on a foundation and a scale" equal to the needs of the city. The Sheriff, Mr. R. H. Swain, endorsed this suggestion, and proposed the erection of a new wing, with "provision for all the memorial tablets that might be desired." Since other schemes have been mooted we hope that the send-off thus courageously given to the plan for an overdue extension of the hospital will be followed by a general approval. A waiting list of 1,000 civil patients is a grave problem, and Mr. Player has only to explain how much two wards of, say, forty beds in all could do to reduce this number for the public to realise, first, the urgency of the need, and, secondly, how a generous movement on its own part could relieve the congestion.

### A RUMOUR DENIED.

We are glad to learn from a letter by Dr. Theodore Beebe, published in the *Morning Post*, that the widely circulated story of German inoculation of French civilians with tuberculosis toxine, upon which we commented in these columns last week, is untrue, or at all events founded on far from sufficient evidence. Dr. Beebe, it appears, interviewed a number of these refugees, who stated that certain of them had been inoculated with something, the nature of which they did not know. Those who were inoculated were suffering from tuberculosis, whilst the others were not. Most of them were, however, aged and infirm, and suffering greatly from the mental anxieties and physical hardships they had undergone, and in consequence unable to give an intelligible account of themselves. Beyond their statements as to the unknown character of the vaccine administered, there is happily nothing to suggest that it was anything of a harmful nature. Apparently, even the Hun, or at all events the German medical faculty, still possess some latent sparks of humanity, though their previous exploits of poisoning wells with bacteria fully justified the *Morning Post* in making the supposition that it did.

### THE AFTER-CARE OF DISCHARGED SOLDIERS.

A committee for dealing with discharged soldiers has presented its report to the Birmingham Citizens' Committee. The scheme contained therein proposes co-operation with the Local Committee and the Employment Exchange so far as employment is concerned. In conformance with the policy of the Statutory Committee cases presenting special difficulty or needing medical treatment will be sent to the Local Committee to deal with, and the men will be informed that the Government wishes them to register at the Employment Exchange. "All refusals on the part of discharged men to accept employment which in the opinion of the manager of the Exchange is suitable, will be reported to the Local Committee for such action as it may deem desirable." This looks as if the men were to be more at the disposal of the Exchange than is likely to be popular with Englishmen, and we hope that public opinion will act as a strong check upon the official autocrat; if not, there is likelihood of grave abuse. Hospitals have been consulted as to the treatment of special cases, and the proposed scale of fees is being now considered. The Orthopædic Hospital is already treating many cases. Discharged men are to be visited systematically by District Committees, which will try "to persuade the men to accept" the training and treatment laid down for them.

### SUICIDE IN WARTIME.

The newspapers frequently give prominence to some case of suicide that may be traced to war conditions—to bereavement or worry. A man kills himself because he is rejected for the Army, or his wife does so because her husband has to join up. Of course such cases attract attention, but the fact is that there has been a considerable decrease in at least one class of suicides since the war. Suicides by poison numbered over 500 in 1913—the last complete peace year, while in 1915—the first complete war year—they were only about 300. This decrease may be due in part to the increased prices of poisons, and to more care in the dispensing of them; but it is quite possible that the morbid-minded, who are perhaps those who incline most to death by poison, have had their attention distracted from their own personal trouble by the pressure of public events. Whatever the cause, the fact of the decrease remains. In 1913 the suicides by hydrochloric acid numbered 98, and in 1915 only 47. Oxalic acid, which is not difficult to obtain, caused 88 deaths the year before the war and only 38 in the first war-year. An almost equal diminution is found in the use of cyanide of potassium and prussic acid, the figures being 77 and 39 deaths respectively. Among the compensations of war it seems that we must place the lessening of that morbid selfishness which makes certain people throw away their lives because all has not gone well with them.

### THE WELSH MEMORIAL AND THE SOLDIERS.

Mr. D. W. Evans, director of the Welsh National Memorial Association, discussed the present position before the Cardiff Health Committee. He led a deputation, the object of which was to ask that the halfpenny rate which the Corporation had agreed to pay should apply to each of the four years of the Association's work. Mr. Evans explained that on account of the war there was a deficiency due to reduced receipts, and also to the greater number of patients treated. This latter point is partly explained by the fact that the Association has undertaken to treat tubercular wounded and discharged soldiers. The result of the request being granted, as it was after discussion, is that Cardiff's contribution will amount to £890. This grant, it must be added, was conditional upon the adoption by other contributing authorities of a similar generous policy. Their refusal to do so would almost amount to an expression of want of confidence in the Association, and we trust that its work for soldiers will receive due recognition.

### PRACTICAL PHILANTHROPY BY PERSONAL SERVICE.

Darning and mending the clothes and socks of the wounded soldiers in hospital is not a very romantic occupation, but it is an absolutely necessary and practical proceeding. Those who undertake to be responsible for the whole of such work for a big hospital are justly entitled to the gratitude of all concerned. At the beginning of the war, Mrs. Trevor Tyler, wife of General Tyler, of Llantrithyd, organised a band of voluntary workers with the object of doing the mending and darning for all the soldier patients at the 3rd Western General Hospital, Cardiff, and ever since then those ladies have carried out the undertaking with a commendable regularity and thoroughness. It is a kind of care that wounded patients badly need in many hospitals, and a work in which voluntary assistance is invaluable.

### PERILS OF TUBERCULOUS MILK.

Medical officers are for ever complaining of the peril of tuberculosis infected milk, but little is done to free herds from tuberculosis, and Government efforts in this direction were postponed through the war. But, meanwhile, the Birmingham Drainage Board, which farms four hundred acres and has a famous herd of dairy cattle, has slowly but surely got rid of tuberculosis, and is now able in its annual report to declare that the cattle are not only free from disease, but to claim that this remarkable result has been achieved without loss. It was on the suggestion of Dr. Robertson, the city's medical officer of health, that the Board started to free the herd of tuberculosis, for then the tuberculosis test gave a reaction of 60 per cent. in the case of cows, and 12 per cent. in the case of heifers. Taking the herd as a whole, the percentage of reaction was 36. The report says that the fact that it took three and a half years to get rid of the disease does not imply that it could not have been done in a shorter time; but both the medical officer of health and the veterinary surgeon for the city advised that the change should be gradual, so as to avoid pecuniary loss and reduction of the quantity of milk which the Board was under contract to supply, and the fact that it was accomplished without loss rendered the object lesson of great value. In this connection the medical officer of health declares that the work accomplished is one that has demonstrated to farmers the possibility of freeing a herd of cattle from tuberculosis without the necessity of constantly buying in expensive tubercle-free stock. Farmers in the Birmingham area have taken up the question of freeing their herds from tuberculosis in a way that has not been done anywhere else in England. It would have been extremely difficult to demonstrate to them the desirability of rearing their own stock and not buying in, but the success obtained by the Drainage Board has been used as an example.

### DEATH OF A VETERAN HOSPITAL SECRETARY.

The death occurred on the 17th inst. of a well-known figure in hospital life in the North—Major Cyril Tyler, a vice-president of Stockport Infirmary and for a number of years assistant secretary and secretary of that institution. Major Tyler at the time of his death was aged seventy-seven, and he had lived an interesting as well as a long life. His military career commenced in 1859 when he joined the Indian Army, and he took part in the Abyssinian expedition against King Theebaw in 1867-68, his services being specially commended by Lord Napier of Magdala. Major Tyler was a member of all the associations for promoting intercourse between hospitals and amongst hospital officers, and up to quite recently was a regular attendant at all meetings and congresses frequented by hospital people. He had a particularly charming personality and will be greatly missed by all who knew him.

### MEMORIAL TO A HOSPITAL TREASURER.

A stained-glass window has been erected in the main corridor of the Royal Lancaster Infirmary to the memory of Lieutenant Spencer E. Barrow, treasurer of the institution. Lieutenant Barrow was a member of the Society of Friends, one of a well-known Quaker family, and when war broke out, though past military age, he was granted a commission in the Royal Lancaster Regiment and went out to France to join the battalion. He only reached the trenches where the battalion was established a few hours before he was wounded by a shell in the left arm. For a time there seemed to be encouraging signs of recovery, but he succumbed to his injuries in November 1915. His remains were removed to Lancaster, and accorded a military funeral, the first member of the Society of Friends to be so honoured. The window is the work of Messrs. Shrigley and Hunt, of London and Lancaster, and is a beautiful piece of workmanship. It represents a Roman soldier with a broken sword underneath his feet, a dove and olive-branch on his shield, and a lamb in his arms, an emblem of "armed peace." The figure is surrounded by rich ornament in white and stained glass. An inscription on a tablet of stained green oak is as follows:—

To the Memory of
Spencer Ellwood Barrow, A.R.I.B.A.,
Lt. 5th Bn. King's Own Royal Lancaster Regt.,
Hon. Treasurer of this Royal Infirmary,
A member of the Society of Friends,
Who died, aged 42 years, on the 16th Nov. 1915,
From wounds received in action,
This window is erected by his mother, brother, sisters, and brother-in-law.

The relatives named also handed to the institution a sum of money (to be invested) in memory of the deceased.

### THE SUPERVISION OF THE CONDITIONS IN WHICH V.A.D. WORKERS LIVE.

A correspondence in the public press has been taking place during the past week concerning the unfavourable hygienic and other conditions with which many V.A.D. workers are obliged to put up in auxiliary and V.A.D. hospitals. Quite probably these complaints have a substantial basis of fact, and they should certainly be looked into and remedied when found to be true. For every such complaint which is made public, or indeed entered officially at all, there are very probably a score which are known to none but the sufferers and their intimate friends; for V.A.D. workers are inspired by so high an ideal of patriotism that most of them regard it as their duty to suffer in silence. In our opinion not enough has been done to ensure that proper accommodation, proper food, proper opportunities for rest and relaxation, and proper conditions of work and life in general shall be ensured for these ladies. The official Army supervision of these Red Cross hospitals confines itself, in our experience, to medical and administrative matters. Nursing matters do not come within the province of the R.A.M.C., and the evidence available rather points to the conclusion that the supervision of nursing conditions in military hospitals is less thorough and less effective than that of the other departments, which is conducted through the Army Medical Service.

### THE OVERWORKED PROBATIONER.

How timely was the warning uttered by Sir Henry Burdett in his article last week concerning the overworking of probationers is well illustrated by a recent speech of Colonel Duncombe, the County Director for the West Riding, in opening the new open-air ward at Liversall Hall V.A.D. Hospital, Doncaster. After stating that the new ward was a model, Colonel Duncombe said that there was no doubt in his mind that all the extra accommodation in the country would be needed, for before July he believed that there would hardly be a single spare bed in our voluntary hospitals. This meant that the work would become very arduous, and staffs would have to be increased or their leisure curtailed. The latter, he added, would be very difficult, because at the present moment everybody in the voluntary hospitals was doing as much work as was really good for her. As our article showed, this is an understatement, and we hope that matters will not have to get worse before they are remedied, as they will have to be if the war lasts for a long period. It must be recognised that probationers are already overworked, and that additional numbers must be added to the staffs if the war hospitals are not, even in time of stress when allowances must be made, to fall below the high standard which they claim. Our young women in the hospitals have a hard time, and to demand more from them can only lead to inefficient service and a number of serious breakdowns.

### A BED ENDOWED BY JOINT FAMILY GENEROSITY.

A practical example of family unity and of a commendable desire to perpetuate the revered memory of the family head comes from Leicester in the announcement that the widow, son, and three daughters of the late Mr. Joseph Frisby have each sent cheques to the Royal Infirmary, the total of which (£1,000) it is desired should be used for the endowment of a bed in the institution. The late Mr. Frisby was for many years successfully associated with the boot and shoe industry of the town, and there can be no better testimony to his family's affection for his memory than the tribute they have paid in endowing the "Joseph Frisby" bed. The instance may well be commended as one worthy of general emulation.

### A COMPETITION FOR SOLDIER PATIENTS.

The current number of the *Craigleith Hospital Chronicle* announces a competition for its patients which stirs the heart of civilians at least. The subject of the competition is "My most thrilling experience during the war." The author of this suggestion is perhaps not imprudent in giving, by way of encouragement, such farcical interpretations of the phrase as that of the young officer who declared that he had experienced nothing so thrilling as seeing a pretty girl acting as an omnibus conductor. The competition, however, is the point for the moment, and we hope it will produce good things, such as the *Chronicle* has already given its readers a taste of. There is some good comic verse, "War-time Wooing," by J. S. M., in the same number, and a readable article on "The Battle of Jutland," as seen from an East Coast naval port by the Rev. W. Stephen.

### A SKETCH IN A "GAZETTE."

Hospital gazettes do not escape from the common troubles of journalism. If they are fortunate in possessing readers who write, and are as certain as other papers to count their contributors among their subscribers, they share the general difficulty of obtaining paper, of the rise in price of material and labour. They, too, have to raise their prices, though, for the reasons given above, they are probably able to contemplate the effect of this rise with equanimity. The *Gazette* of the 3rd London General Hospital, London, has published its April number at the higher price of fourpence. Pte. J. H. Dowd's drawings are perhaps the best, but Sergt. Noel Irving's sketch of a man (C 3) ploughing the "sacred tennis-courts" is worth mention, if only because the tennis-courts appear to be situated on the brow of a hill, which slopes so gracefully as to hide, one supposes, the players from each other. We indulge in this commonplace criticism from mere captiousness, since the illusion of a retired and romantic spot trampled by heavy horses and wrinkled by the ploughshare is successfully given, and what does it matter if the site is called a tennis-court or not? A drawing is more important than its title.

### THE COST OF AFTERNOON TEA IN HOSPITALS.

At one large charitable institution located in an outlying suburb of London, a charge of threepence has been made for some time to each patient's visitor who desires to take tea. Later on, owing to war conditions, the charge of threepence was advanced to fourpence, and for this sum a cup of tea and two slices of bread and butter are allowed. Lately, some of the visitors have touched the electric bell and calmly requested the nurse to supply them with more bread and butter. The position was a difficult one for the nurse, who naturally wished to keep on friendly terms with the patients and the patients' visitors; but the question has been solved by the house committee, who have passed a resolution to the effect that any patient's visitor taking tea at the hospital shall be limited to two slices of bread and butter. We think this decision is a wise one, for there is really no call upon a voluntary charity to spend money on feeding the visitors of patients under treatment. We do not say that any profit should be made over the transaction, but, at any rate, the hospital finances should be entirely safeguarded from loss.

### SHOULD BAD MEAT BE STERILISED?

Germans sterilise condemned meat and sell it to the poor. Sundry authorities in England have lately been inquiring into the possibility of such arrangements. The City of London, which has thousands of tons of bad meat from the Smithfield Market to deal with, has decided against treating it so as to enable it to be used for human food. Manchester Corporation has investigated the problem, and with a similar result, but proposes a scheme for using condemned meat for industrial purposes. The inquiry committee reports that it is satisfied that although most of the condemned meat might profitably be used for industrial purposes, very little, if any, could be utilised for food without an alteration of the existing law. Even if the law were altered, the committee is convinced that British prejudices would prevent such schemes as those popular in Germany being profitable over here. Nevertheless it is significant that the Health Committee of the Dundee Corporation has agreed to open a depôt for the sale of sterilised meat, and Captain Leighton, of the Local Government Board, is reported to have declared that the process for the treatment of condemned meat would make it absolutely safe.

### THIS WEEK'S DRUG MARKET.

The advancing tendency of prices continues to be the characteristic feature of the drug market, and with one or two exceptions practically all the changes are in an upward direction. Sodium salicylate is rather scarce at the moment, and higher prices are asked; very little of the drug is arriving from the United States. There is a good demand for antifebrin, and there is a likelihood of the product becoming dearer. Phenacetin is scarcer and in good demand. Resorcin is less scarce than it was. Bromides have an upward tendency in price. Both citric acid and tartaric acid are again dearer. Castor oil continues to advance in price. Business has been done in colchicum corm at a high figure. Gentian root continues scarce. A fair business is being done in bismuth preparations at normal rates. Liquid paraffin is difficult to obtain, and prices have advanced substantially.

